# Haploidentical Stem Cell Transplantation in Children With Hematological Malignancies Using αβ^+^ T-Cell Receptor and CD19^+^ Cell Depleted Grafts: High CD56^dim^/CD56^bright^ NK Cell Ratio Early Following Transplantation Is Associated With Lower Relapse Incidence and Better Outcome

**DOI:** 10.3389/fimmu.2019.02504

**Published:** 2019-10-30

**Authors:** Miguel A. Diaz, Josune Zubicaray, Blanca Molina, Lorea Abad, Ana Castillo, Elena Sebastian, Eva Galvez, Julia Ruiz, Jose Luis Vicario, Manuel Ramirez, Julian Sevilla, Marta González-Vicent

**Affiliations:** ^1^Hematopoietic Stem Cell Transplantation and Cellular Therapy Unit, Department of Pediatrics, Hospital Infantil Universitario “Niño Jesus”, Madrid, Spain; ^2^Blood Bank and Graft Manipulation Unit, Division of Hematology, Hospital Infantil Universitario “Niño Jesus”, Madrid, Spain; ^3^Oncology/Hematology Laboratory, Hospital Infantil Universitario “Niño Jesus”, Madrid, Spain; ^4^Histocompatibility Laboratory, Community Transfusion Center of Madrid, Madrid, Spain

**Keywords:** T-cell depletion, NK cells, haploidentical transplantation, immune reconstitution, acute leukemia, children

## Abstract

We prospectively analyzed outcomes of haploidentical hematopoietic stem cell transplantation using αβ^+^ T-cell receptor/CD19^+^ depleted grafts. Sixty-three transplantations were performed in 60 patients. Twenty-eight patients were diagnosed with acute lymphoblastic leukemia (ALL), 27 patients were diagnosed with acute myelogenous leukemia, and in eight other hematological malignancies were diagnosed. Twenty-three were in first complete remission (CR), 20 in second CR, 20 beyond second CR. Four patients developed graft failure. Median time to neutrophil and platelet recovery was 14 (range 9–25) and 10 days (range 7–30), respectively. The probability of non-relapse mortality (NRM) by day +100 after transplantation was 10 ± 4%. With a median follow-up of 28 months, the probability of relapse was 32 ± 6% and disease-free survival was 52 ± 6%. Immune reconstitution was leaded by NK cells. As such, a high CD56^dim/^CD56^bright^ NK cell ratio early after transplantation was associated with better disease-free survival (DFS) (≥3.5; 77 ± 8% vs. <3.5; 28 ± 5%; *p* = 0.001) due to lower relapse incidence (≥3.5; 15 ± 7% vs. <3.5; 37 ± 9%; *p* = 0.04). T-cell reconstitution was delayed and associated with severe infections after transplant. Viral reactivation/disease and presence of venooclusive disease of liver in the non-caucasian population had a significant impact on NRM. αβ^+^ T-cell receptor/CD19^+^ cell-depleted haploidentical transplant is associated with good outcomes especially in patients in early phase of disease. A rapid expansion of “mature” natural killer cells early after transplantation resulted on lower probability of relapse, suggesting a graft vs. leukemia effect independent from graft-vs.-host reactions.

## Introduction

Nowadays, allogeneic hematopoietic stem cell transplantation is considered a curative option for pediatric patients with high-risk leukemia at diagnosis or after relapse. However, an important proportion of those patients lack a suitable human leucocyte antigen (HLA) match donor or delay for donor availability may be unacceptable (1). For patients needing an allogeneic transplant in a timely manner, the use of haploidentical relative donors has been considered a suitable approach (2, 3). Donor T-cells in the graft facilitate T-cell reconstitution but they are also responsible for graft-vs.-host disease (GvHD). Currently, several haploidentical transplant platforms using “*ex-vivo*” T-cell depletion (TCD) without using post-transplant GvHD pharmacological prophylaxis have gained clinical relevance (4, 5).

Natural killer (NK) cells are have been detected in the first weeks following TCD haploidentical transplantation and the pattern of expressed killer cell immunoglobulin-like receptors (KIRs) was similar to that originally found in the donor (6). However, NK cell phenotype was altered and cytotoxicity was lower compared with their donors in pediatric patients receiving CD3^+^/CD19^+^ depleted haploidentical transplantation (7). The mentioned TCD haploidentical transplantation platform was associated with encouraging results especially in patients in the early phase of disease (8). KIR-B haplotype donors conferred a rapid NK cell expansion early after transplant, which resulted in lower probability of relapse. Using grafts from younger donors resulted in an improved immune reconstitution in all lymphocyte subsets (9) however, viral reactivation affected a significant proportion of patients.

A new approach to improve delayed immune reconstitution has been recently developed, based on selective depletion of αβ^+^ T lymphocytes, and of B cells (10). Previous T-cell depleted strategies resulted in loss of lymphocyte cell subsets that may play a positive role in the recipient. T cells displaying the αβ T-cell receptor (TCR) are responsible for GVHD. However, T cells with the γδ receptor chains have no alloreactive capacity, and may contribute to important anti-infectious activity and to a possible anti-leukemia effect. A recent publication using the mentioned haploidentical transplant platform are mainly focused on early immune reconstitution especially on γδ^+^ T cells subsets (10).

However, few studies have reported a detailed analysis of prognostic factors and outcomes in this type of transplantation considering early immune reconstitution and its impact on transplant outcomes (10, 11). The aim of this observational prospective study was to analyze the outcomes and risk factors for survival of pediatric patients who received an haploidentical relative donor transplant using αβ^+^ TCR/CD19^+^ depleted grafts.

## Patients, Materials and Methods

### Patient and Transplant Characteristics

Patients were enrolled in the study from November 2012 to November 2018.

All consecutive patients, younger than 20 years old, diagnosed with high-risk hematological malignancies, in need of an allogeneic transplantation, and in good clinical condition who lacked either a matched related donor or a matched unrelated donor, were included. Indications for allogeneic hematopoietic transplantation for children with acute lymphoblastic leukemia (ALL) included: poor cytogenetics, induction failure (defined as no remission at 1 month following induction treatment), persistent minimal residual disease tested by PCR before transplant with a cutoff point of 10^−3^ and 2nd complete remission (CR) or beyond. For acute myeloid leukemia (AML) patients transplant criteria included intermediate or poor risk characteristics at diagnosis and also 2nd CR or beyond. Primary refractory AML was defined as the failure to achieve a response after one or two cycles of induction. Refractory ALL was defined as the failure to achieve a cytological remission response after induction chemotherapy and second-line rescue chemotherapy. The only exclusion criterion was a poor clinical condition, defined as a Lansky score lower than 60%.

Patients, parents, and/or their legal guardians gave written informed consent in accordance with the Helsinki Declaration.

Sixty-three transplants were performed in 60 patients. Twenty-eight patients had ALL, 27 AML, five advanced myelodysplastic syndrome (MDS), two Hodgkin disease, and one non-Hodgkin lymphoma. Twenty-three were in first CR, 20 in second CR and 20 in more than second CR or active disease at time of transplantation. Twenty-two patients had MRD positive at time of transplant. Haploidentical was the first transplant in 49 cases. In 14 cases, one or two previous stem cell transplantations and subsequent chemotherapy courses before haploidentical transplantation were performed.

Patient and transplantation characteristics are shown in Table 1.

**Table 1:** Patient, donors, and transplant characteristics.

**Number**	63
**Patient age, years**	
Median (range)	9 (1–19)
**Patient weight, kg**	
Median (range)	30 (8–136)
**Patient gender**	
Male	41 (65%)
Female	22 (35%)
**Patient lansky score**	
90–100	55 (87%)
<90	8 (13%)
**Patient CMV serostatus**	
Positive	40 (64%)
Negative	23 (36%)
**Patient ethnicity**	
White caucasian	47 (75%)
White non-caucasian (Hispanic/North Africans)	16 (25%)
**Disease**	
Acute myeloid leukemia	27 (43%)
Acute lymphoblastic leukemia	28 (44%)
Myelodisplastic syndrome/refractory cytopenia childhood	5 (8%)
Hodgkin disease	2 (3%)
Non-Hodgkin Lymphoma	1 (2%)
**Disease status at transplant**	
1st CR	23 (36%)
2nd CR	20 (32%)
3rd CR or active disease	20 (32%)
**Minimal residual disease status**	
Positive	22 (35%)
Negative	41 (65%)
**Donor age, years**	
Median (range)	40 (10–54)
**Donor gender**	
Male	27 (43%)
Female	36 (57%)
**Donor CMV serostatus**	
Positive	42 (67%)
Negative	21 (33%)
**Donor KIR genotype**	
KIR B	58 (92%)
KIR A	5 (8%)
**Donor KIR B score**	
Neutral	30 (52%)
Better	21 (36%)
Best	7 (12%)
**KIR ligand-ligand matching**	
Match	33 (52%)
Mismatch	30 (48%)
**Transplant number**	
1^st^ transplant	49 (78%)
2^nd^ transplant	10 (16%)
3^rd^ transplant	4 (6%)
**Graft composition**	
CD34^+^ cells × 10^6^/Kg median (range)	7.59 (2.06–16.56)
CD3^+^ cells × 10^6^/Kg median (range)	5.89 (1.30–46.26)
CD3^+^ TCR*αβ* cells × 10^5^/Kg median (range)	0.01 (0.01–0.78)
CD3^+^ TCR*γδ* cells × 10^6^/Kg median (range)	5.64 (0.13–46.17)
CD3^−^CD56^+^ cells × 10^6^/Kg median (range)	32.20 (0.18–139.54)
CD3^−^CD19^+^ cells × 10^5^/Kg median (range)	0.04 (0.01–1.34)
Median follow-up of survivors, months (range)	28 (4–72)

### KIR Genotyping and KIR Ligand

Fifteen human KIR genes and two pseudogenes were analyzed by PCR with a KIR typing kit (Miltenyi Biotec, Bergisch Gladbach, Germany). The KIR A haplotype was defined by the absence of 2DS1, 2DS2, 2DS3, and 3DS1 and the presence of 2DS4 as the only KIR-activating receptor. The KIR B haplotype was determined by the presence of any activating genes except 2DS4. The KIR ligand HLA-C allotypes (C1 and C2) and the HLA-B allotypes (Bw4) were determined using high-resolution PCR-sequence-based typing.

We also determined KIR B-content scores for all donors according to the system proposed by Cooley et al. (12) (www.ebi.ac.uk/ipd/kir/donor_b_content.html). Criteria for donor selection have been previously reported (8, 13). Briefly, donors were chosen based on KIR B haplotype, higher B-content score, younger age, and NK alloreactivity (KIR-Ligand model). Donors were parents (mother in 34 and father in 27) or siblings in 2. Donor characteristics are also shown in Table 1.

### Donor Hematopoietic Stem Cell Mobilization, Collection, Graft Manipulation Procedure and Infusion

Donor mobilization has been previously described (8, 9, 14). Briefly, mobilization started on day 5 of the conditioning regimen at a G-CSF dose of 10 μg/kg/day subcutaneously. Based on the volume, the dose may be split into two injection sites. Progenitor cells collections were performed by leukapheresis. In all, 66 products were obtained by large-volume leukapheresis procedure according to established protocols of the center using a continuous flow blood cell separator (Spectra Optia MNC v.3.0. Terumo BCT, Lakewood, CO; USA or COBE Spectra TM, v.6.1, by Caridian BCT Europe, Garching, Germany) on the fifth day of mobilization and the day before infusion. Apheresis was carried out via bilateral peripheral veins whenever possible, or otherwise by a central venous catheter. During leukapheresis, between 3 and 5 blood volumes were processed. Acid citrate dextrose (ACD-A) was used as an anticoagulant with a ratio of 14:1. Leukapheresis products were also analyzed for expression of the CD34^+^ antigen as previously reported (8). Concurrent plasma (200–300 mL), was collected for products to be stored overnight after receipt into the processing facility. A unique identification and labeling system has been used to track leukapheresis product from collection to infusion according to FACT/JACIE guidelines. A target dose ≥5.0 × 10^6^ CD34^+^ cells/kg after selection containing ≤25.0 × 10^3^ CD3^+^ αβ^+^ TCR cells/kg was desired. If after two collections, the minimum required dose CD34^+^ cell dose (>2.0 × 10^6^ per kg) were reached, no more collections were performed.

T-cell depletion was performed using CliniMACS Plus device or the fully automated Prodigy device after manipulations in a laminar-flow cabinet located in a clean room certified for sterile manipulations. Clinical grade reagents, disposable kits, and instrumentation were from Miltenyi Biotec (Bergisch Gladbach, Germany).

Before depletion procedure, cell composition of the apheresis product were analyzed by flow cytometry (cell count and cell subpopulation CD34^+^, CD3^+^ αβ^+^, CD3^+^ γδ^+^, CD19^+^, and CD3-CD56^+^ cells, and theirs viability), to adjust the doses of total nucleated cells (TNC) and αβ^+^ T cells to the maximal capacity of <60 × 10^9^ TNC and <24 × 10^9^ αβ^+^ T-cells in case of CliniMACS system (CliniMACS system, Miltenyi Biotec, Germany), or <45 × 10^9^ TNC and <20 × 10^9^ αβ^+^ T-cells in case of Prodigy device (Prodigy system, Miltenyi Biotec, Germany), according to the manufacturer's instructions.

We use the CliniMACS Prodigy device together with the CliniMACS Prodigy TS310, CliniMACS TCR αβ -biotin reagent, CliniMACS Anti-Biotin reagent, CliniMACS CD19 reagent, and CliniMACS PBS/EDTA, all of them obtained from Miltenyi Biotec GmbH (Bergisch-Gladbach, Germany).We selected the process “LP TCT αβ−19 Depletion” normal scale; this process uses the following reagents: CliniMACS TCR αβ -biotin reagent, CliniMACS Anti-Biotin reagent, CliniMACS CD19 reagent (1 vial of each), and CliniMACS PBS/EDTA; a CliniMACS TS310 tubing set was installed and the liquids were connected as prompted by the device. The cells passed through a magnetic field through which they will be separated. Thus, αβ^+^ T and CD19^+^ lymphocytes are removed/eliminated/retained, while all unlabeled cells are recovered for subsequent transplantation to the patient.

At the end of the procedure, quality control by flow cytometry was carried out in the final product, such as cell counts, viability studies, and aerobic and anaerobic cultures before and after immunomagnetic depletion.

In all, 66 depletion procedures were performed (48 out of by the CliniMACS Plus system (72.72%) and 18 depletions were performed with CliniMACS Prodigy device (27.27%) according to the availability of both devices. None of the apheresis products exceeded the maximal capacity/limits of TNC or αβ^+^ T cells established by the manufacturer at the beginning of the manipulation procedure. Median recovery of CD34^+^ were 81.92% with CliniMACS Plus and 73.70% with CliniMACS Prodigy, without reaching statistical significance. There were no significant differences in terms of the T cells depletion efficacy assessed by logarithmic descent (4.41 vs. 6.03, *p* = 0.42). The median infused doses of αβ^+^ T cells/kg cells were 3.89 × 10^3^ and 2.18 × 10^3^ in each group. However, there was a higher number of infused γδ^+^ T-cells/Kg recipient body weight using CliniMACS Prodigy (71.90 × 10^3^/kg) vs. CliniMACS Plus 15.13 × 10^6^/kg (*p* = 0.015). Data on cell composition of final apheresis products after depletion procedures using both systems are provided in Supplemental Table 1. All cellular products were freshly infused on day 0.

### Transplantation Protocol

The conditioning regimen consisted of fludarabine (25 mg/m^2^ day from days −6 to −2), busulfan (3.2–4.8 mg/kg/day, according to patient body weight, from days −6 to −4) and thiotepa (5 mg/kg/day from days −3 to −2). Methylprednisolone was administered on days −6 to −2 (5 mg/kg). No serotherapy was given as part of transplant conditioning.

For those patients who required a second haploidentical transplant due to primary graft failure, the conditioning regimen used consisted of fludarabine 40 mg/m^2^/day from days −5 to −3, thymoglobulin 2 mg/kg/day from days −5 to −3, and melphalan 120 mg/m^2^ on day −1. Cyclosporine was used as pharmacological GvHD prophylaxis from day −1 until engraftment and it was tailored as soon as possible after transplant whenever acute GvHD was not present.

### Assessment of Engraftment, Chimerism Status, Antibodies and Flow Cytometry Analysis for Immune Reconstitution Following Transplantation

Myeloid recovery was defined as the first of 3 consecutive days on which the absolute neutrophil count was ≥0.5 × 10^9^/L. Platelet recovery time was considered the first of 3 consecutive days on which a platelet count above 20 × 10^9^/L was achieved, with no transfusion requirements.

Donor chimerism was evaluated by the short tandem repeat PCR method at the time of engraftment and monthly following transplantation or when clinical condition required. Donor chimerism was determined for whole blood and cell subsets. For chimerism analysis in sub-populations, CD34^+^ progenitor cells from bone marrow, and T lymphocytes from peripheral blood were purified by immunomagnetic methods (CD34 or CD3 beads; Miltenyi Biotec). Phenotyping of NK cells, T lymphocytes, T lymphocyte subsets, B lymphocytes, NKT lymphocytes, and dendritic cells (DCs) was performed on fresh samples of whole blood by multiparametric flow cytometry as previously described (7, 8).

The following fluorochrome-labeled monoclonal Abs against human Ags were obtained from Becton Dickinson (San Jose, CA, USA): CD3 PE-Cy7, CD20-PE, CD45-FITC, lineage-FITC, HLADR-APC-Cy7, CD11c-PECy5, CD45RAPE-Cy5, and CCR7-PE. Fluorochrome-labeled monoclonal Abs against CD19-PE, CD56-APC, and CD25-PE were obtained from Beckman Coulter (Fullerton, CA, USA). Fluorochrome-labeled monoclonal Abs against BDCA4-APC were obtained from Miltenyi Biotec.

### Study Design, Definitions and Statistical Analysis

The major study endpoints were disease-free survival (DFS), cumulative incidence of relapse and non-relapse mortality (NRM). DFS was defined as time from transplantation to relapse/progression or death for any cause, whichever occurred first.

Acute GvHD (aGvHD) was graded according to standard criteria, whereas chronic GvHD (cGvHD) was defined as mild, moderate and severe according to NIH criteria (15) for chronic GvHD. Relapse was defined as morphologic or clinical evidence of recurrence in the peripheral blood, bone marrow, or extramedullary sites. NRM was defined as any cause of death other than disease. The probability of DFS was calculated from the time of transplantation by means of the Kaplan–Meier product limit method (16).

Cumulative incidence was used to estimate the relapse and NRM probabilities (17). Death in remission was treated as a competing event to calculate the cumulative relapse incidence. Relapse was considered to be the competing event for calculating cumulative incidence of NRM. Gray's test was used to assess differences between relapse incidence and NRM. The multivariate analysis of survival was evaluated using proportional hazard regression model (18). Hazard ratios (HRs) were calculated with a 95% confidence interval (95% CI). A *P* < 0.05 was considered statistically significant. The following variables were included in the analysis as covariates: patient and donor age, diagnosis, diseases status at time of transplantation, cell graft composition, KIR ligand mismatch status, donor KIR haplotype, KIR B score, and kinetics of immune reconstitution. End points were calculated at the time of last contact, loss of follow-up or death. Statistical analyses of data were performed using statistical package SPSS for Macintosh (software version 20.0; IBM Corporation, Armonk, NY, USA) and the R software package for Macintosh (version R 3.3.3).

## Results

### Engraftment Kinetics, Supportive Care and Toxicity Profile

Four patients developed primary graft failure. All of them were rescued using a second allogeneic transplant. The median time to neutrophil recovery for engrafted patients was 14 days (range; 9–25). The median time to platelet engraftment was 10 days (range; 7–30). Data on engraftment kinetics are provided in Table 2. All patients achieved full donor chimerism at time of engraftment. Patients required platelet transfusions for a median time of 2 days (range; 0–54) and the median duration of the red blood cell transfusions was 2 days (range; 0–20). Data on supportive care are also provided in Table 2.

**Table 2:** Engraftment and supportive care.

**Outcomes**	**Value**
**Engraftment (on days)**	**Median (range)**
Time to neutrophils	14 (9–25)
Time to platelet ≥ 20 × 10^9^/L	10 (7–30)
Time to platelet ≥ 50 × 10^9^/L	13 (7–35)
Time to platelet ≥ 100 × 10^9^/L	16 (9–50)
**Supportive care (on days)**	
Time on fever	1 (0–12)
Time on antibiotics treatment	18 (3–85)
Time on RBC transfusion	2 (0–20)
Median time on platelets transfusion	2 (0–54)
Length of hospital stay	15 (10–86)

The toxicity profile is shown in Table 3. Seventeen patients (27%) developed engraftment syndrome (ES). Seven patients (11%) developed veno-occlusive disease/sinusoidal obstruction syndrome (VOD/SOS) of liver.

**Table 3:** Transplantation-related toxicity.

**Toxicity**	
**Mucositis**	***N*** **=** **41**
Grade 1–2	28 (44)
Grade 3–4	13 (21)
**Nausea and vomiting**	***N*** **=** **23**
Grade 1–2	10 (16)
Grade 3–4	13 (21)
**Diarrhea**	***N*** **=** **25**
Grade 1–2	15 (24)
Grade 3–4	10 (16)
**Organ toxicity (grade 3**–**4)**	***N*** **=** **12**
Hepatic (including VOD/SOS)	7 (11)
Cardiac	0 (0)
Neurologic	4 (6)
Renal	1 (2)
Pulmonary	0 (0)
**Engraftment syndrome**	17 (27)
**Thrombotic microangiopathy**	3 (5)

This complication was significantly associated to ethnicity. Incidence of VOD/SOS was 4% in Caucasians whereas in non-Caucasians rose to 29% (OR 9.17, range 1.58–52.6; *p* = 0.005). The median hospitalization time after hematopoietic cell infusion was 15 days (range; 10–85).

### Immune Reconstitution

The immune reconstitution was characterized in all patients with engraftment by a rapid increase in NK cells. The NK cell counts peaked on day +30 [median of 250/μL (range; 1–2,046)]. The median number of CD56^dim^ NK cells on day +30 was 210/μL (3–2,040) whereas the median number of CD56^bright^ NK cells on day +30 was 40/μL (10–350). The median CD56^dim^/CD56^bright^ NK cell ratio was 3.5 on day +30 (range; 1–45). After day 30, the NK cell population slowly decreased, being a median of 185 (8–1,381) cells/μL on day +180. Ultimately, the NK cell count remained steady at ~160–180 cells/μL.

T-cell recovery was delayed with a median number of 155 CD3^+^ cells/μL (range, 11–1,694) on day +30, 185 cells/μL (range, 3–4,740) on day +60, 293 cells/μL (range, 5–6,250) on day +90, and 490 cells/μL (range, 18–3,250) on day +180.

There was a relationship between the infused CD34^+^ cell dose and T-cell recovery (r = 0.540; *p* = 0.03). The reconstitution of B lymphocytes was also delayed, starting with a median of 2 cells/μL (range, 0–414) on day +30 and progressively increasing until reaching 90 (range, 2–4,927) on day +90, and 240 (range, 2–9,99) on day +180. We observed a gradual increase in the DC population over time, with a median of 20 cells/μL (range, 2–760) on day +30 and 34 (range, 2–270) on day +180. Detailed data of lymphoid subpopulations recovery and kinetics are shown in Supplemental Table S2 and Supplemental Figure S1.

### Acute and Chronic GvHD

Thirty-five patients developed aGvHD (grade 1, *n* = 10; grade 2, *n* = 7; grade 3, *n* = 10; and grade 4, *n* = 8). The probability of aGvHD (grade 2 or higher) was 34 ± 7%. The median time to aGvHD was 45 days (range, 10–90 days). Fourteen patients developed cGvHD (mild in six cases, moderate in six cases, and severe in six cases), with a probability of 25 ± 6%. The median time to cGvHD was 110 days (range, 76–306 days). We found no variables associated to develop neither acute nor chronic GvHD.

### Non-relapse Mortality

With a median follow-up of 28 months (range; 4–72) the cumulative incidence of NRM was 23 ± 5%. Seven patients died by day +100 and 5 at a later time with a median time to death of 86 days (range, 27–216). Univariate analysis revealed that NRM was influenced by ethnicity (white caucasians 14 ± 5% vs. non-caucasians 58 ± 15%; *p* < 0.001); presence of SOS (yes 85 ± 10% vs. no 14 ± 5%; *p* = 0.0003); development of severe aGvHD (yes 66 ± 12% vs. no 5 ± 3%; *p* = 0.0002); and CD56^dim/^CD56^bright^ NK cell ratio on day +30 (low 37 ± 9% vs. high 7 ± 5%). However, multivariate analysis showed that presence of SOS was the only variable associated with NRM (HR: 95% CI; 6.6; 1.1–40.24 *p* = 0.04). The primary causes of death are shown in Table 4.

**Table 4:** Causes of death.

**Cause**	***N* = 22 (%)**
Relapse	11 (50%)
Infections	5 (23%)
Severe VOD/SOS	2 (9%)
TA-TMA	2 (9%)
Refractory GvHD	2 (9%)

### Subset Analysis of Infection Complications Infection Complications

In all, 72 infectious episodes were diagnosed using microbiological and/or clinical criteria (Table 5). All patients experienced at least one infection, with a median of 1 events/patients (1–3) and a median time onset to first infection episode of 13 days (6–45). Median time to first infection was 13 days for bacterial, 25 days for viral, and 20 days for fungal infections. Early immune reconstitution (on day +30) was associated with severe infection defined as sepsis, viral disease, and/or invasive fungal infection. So, patients who developed severe infection had lower counts of NK cells and γδ T-cells (median 122, range; 1–332; and 11, range; 1–77, respectively) compared to those patients who did not develop severe infection episode (median 230, range; 1–1,962; *p* = 0.018 and 34, range; 1–573; *p* = 0.02). However, on day +60, severe infection was associated to low counts of CD8^+^ T-cells (median 23, range 0–51) compared to patients with no severe infection (median 56, range 0–759) (*p* = 0.009).

**Table 5:** Infectious complications.

**Total number of infection episodes**	72
**Bacterial infections**	33 (46%)
**Gram-negative bacilli**	8 (24%)
*Enterobacteriaceae* species	5 (62%)
*Pseudomonadaceae* species	2 (25%)
*Campylobacteraceae* species	1 (13%)
**Gram-positive bacilli**	8 (24%)
*Clostridium* species	8 (100%)
**Gram-positive cocci**	17 (51%)
*Staphylococcus* species	14 (82%)
*Streptococcus* species	2 (12%)
*Enterococcus* species	1 (6%)
**Viral infections**	32 (44%)
CMV	5 (16%)
Adenovirus	4 (12%)
HHV-6	6 (19%)
VZV	3 (9%)
Rinovirus	3 (9%)
Coronavirus	2 (6%)
SRV	3 (9%)
Other viruses	6 (19%)
**Fungal infections**	7 (10%)
*Candida* species	4 (57%)
*Aspergillus* species	3 (43%)

Detailed data regarding bacterial, viral, and fungal infections are provided in Table 5.

### Relapse Incidence

With a median follow-up for survivors of 28 months (range; 4–72 months), 15 patients relapsed, with a median time to relapse of 130 days (range; 37–265 days), and a cumulative incidence of 27 ± 6%. The following variables influenced the probability of relapse in the univariate analysis: disease phase at transplantation (early phase, 5 ± 5%; vs. advanced phase, 37 ± 8%; *p* = 0.01), high CD56^dim/^CD56^bright^ NK cell ratio on day +30 (≥3.5; 15 ± 7% vs. <3.5; 37 ± 9%; *p* = 0.04) and type of acute leukemia (myeloid 11 ± 6% vs. lymphoid 42 ± 9%; *p* = 0.006). Multivariate analysis (MVA) showed that relapse was influenced by type of malignancy and CD56^dim/^CD56^bright^ NK cell ratio on day +30 after transplantation Table 6.

**Table 6:** Multivariate analysis of relapse.

**Factors**	**HR (95% CI)**	***p*-value**
Type of malignancy		
Myeloid	Reference	
Lymphoid	8.8 (2.39–32.45)	0.001
CD56^dim/bright^ NK cell ratio		
High	Reference	
Low	3.75 (1.17–12.03)	0.02

### Disease-Free Survival (DFS)

**Figure 1:**
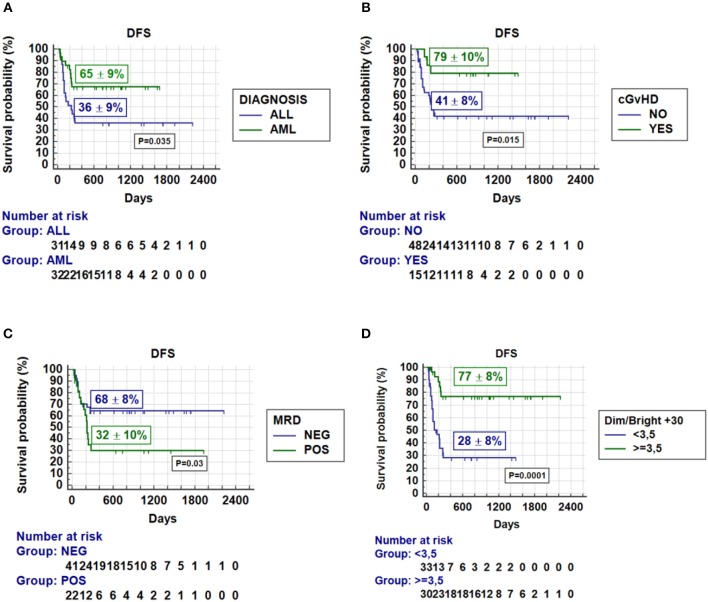
Probability of disease-free survival according to multivariate analysis. **(A)** diagnosis; **(B)** chronic GVHD; **(C)** minimal residual disease status; **(D)** NK^dim/bright^ ratio on day +30.

The probability of DFS was 52 ± 6% for the whole group. The following variables influenced DFS in the univariate analysis: Ethnicity (caucasians 59 ± 7% vs. non-caucasians 15 ± 11%, *p* = 0.006), disease phase at transplantation; early vs. intermediate vs. advanced (81 ± 9% vs. 41 ± 11% vs. 29 ± 11%, respectively; *p* < 0.001), MRD status (negative 68 ± 8% vs. positive 32 ± 11%; *p* = 0.03), type of malignancy; myeloid vs. lymphoid (65 ± 9% vs. 36 ± 9%, respectively; *p* = 0.035), presence of VOD/SOS (no 56 ± 7% vs. yes 14 ± 12%; *p* = 0.004), severe aGvHD (no 62 ± 8% vs. yes 28 ± 11%; *p* = 0.03), chronic GvHD (no 41 ± 8% vs. yes 79 ± 11%; *p* = 0.015), and CD56^dim/^CD56^bright^ NK cell ratio on day +30 (≥3.5; 77 ± 8% vs. <3.5; 28 ± 5%; *p* = 0.001). Variables associated to DFS in MVA are given in Table 7. DFS curves according to MVA are shown in Figure 1.

**Table 7:** Multivariate analysis of DFS.

**Factors**	**HR (95% CI)**	***p*-value**
Type of malignancy		
Lymphoid	Reference	
Myeloid	32.37 (4.25–121.07)	0.0001
EMR status pre-alloHCT		
Positive	Reference	
Negative	5.03 (1.20–21.03)	0.025
Chronic GvHD		
No	Reference	
Yes	20.73 (3.16–136.19)	0.002
CD56 ^dim/bright^ NK cell ratio		
Low	Reference	
High	6.02 (1.62–22.44)	0.007

## Discussion

Allogeneic hematopoietic stem cell transplantation from haploidentical donors is a suitable transplant option for patients lacking a HLA-identical donor. Nowadays, several haploidentical transplant platforms have gained clinical relevance using “*ex-vivo*” T-cell depletion (TCD) (19). In TCD haploidentical setting, different techniques of graft manipulation have been developed over last 15 years to avoid or minimize undesirable GvHD (19–21). These techniques have evolved from the initial almost total T and B cells depletion by means of CD34^+^ isolation and CD3^+^/CD19^+^ depletion to more recently graft manipulation techniques such as αβ^+^ TCR/CD19^+^ or CD45RA^+^ depletions that resulted in partial TCD maintaining NK cells in an attempt to improve immune reconstitution following transplantation (22, 23). An approach to circumvent delayed immune recovery represented by a more sophisticated method of graft manipulation that has been recently developed and implemented is based on selective depletion of αβ^+^ T-cells and B-cells (10). This strategy allows not only the infusion of donor hematopoietic stem cells, but also committed hematopoietic progenitors, mature NK cells, and γδ^+^ T-cells. Recently, haploidentical transplant after αβ^+^ T-cell/B-cell depletion has shown to be effective in single-center studies (10). In these studies, myeloablative total body irradiation (TBI) conditioning regimens combined with serotherapy have been used. Other reported studies used chemotherapy-based conditioning regimens even with no serotherapy as part of conditioning (7–9, 11). Children with acute leukemia receiving haploidentical transplant using αβ^+^ T-cell/B-cell depletion had a relatively lower risk for acute and chronic GVHD even than those transplanted from an unrelated donor. However, major challenges were relapsing disease and delayed immune reconstitution leading infectious morbidity and mortality (11). So, immune reconstitution studies and risk factors for transplant outcome analysis using this TCD haploidentical platform are needed. Herein, we reported the risk factors analysis and a detailed analysis of immune reconstitution and its impact on transplant outcomes in a prospective study using haploidentical transplant with αβ^+^ T-cell/B-cell depletion after chemotherapy-based conditioning regimen with no serotherapy.

The main findings of present study are commented below.

First, engraftment kinetics were fast, probably due to the large number of infused progenitors (Tables 1, 2), as has been recently published (8, 10, 11, 24). As other authors (10, 20, 21) have noted, grafts so depleted, contain high numbers of graft-facilitating cells and CD34^+^ cells, and result on enhanced engraftment and immune recovery. Graft failure was lower than reported (8) with other previous graft manipulation procedures using the same conditioning regimen and very similar to other published studies using TBI-based conditioning with or without serotherapy as part of preparatory regimen. Non-radiation-based preparative regimens have be used in pediatric patients with high-risk acute leukemia and busulfan-based myeloablative conditioning regimens have been reported safe and effective in hematopoietic transplantation (11). An important observation in this study was the fact that the conditioning regimen was well-tolerated (Table 3), with a low incidence of severe complications, similar to that of recently published in adults (25) and children (7–9). However, we have observed a high incidence of severe VOD/SOS in non-Caucasian population not previously observed and reported. This complication was associated with high mortality in the mentioned population. Non-Caucasian (mainly white Hispanic) patients are being increasingly included in the transplant program at our institution. It is possible that there is an interethnic variability in genetic polymorphisms frequencies for metabolizing enzymes that probably influence toxicity incidence such as VOD/SOS. Based in our findings, ethnicity should be considered as potential risk factor for further prospective VOD/SOS studies.

The incidence of ES is remarkable (Table 3). ES is an inflammatory condition that occurs after HSCT and it is characterized by a non-infectious fever and skin rash. We have previously reported (8) this complication in pediatric haploidentical transplantation setting, using other TCD platform. ES has been considered as a herald of GvHD and both complications might be related with not using serotherapy as part of preparatory regimen. However, this complication had no impact on clinical outcomes in our series (8).

Second, immune reconstitution was preceded by a rapid and sustained appearance of NK-cells (Supplemental Table 2). Immune reconstitution after allogeneic transplantation depends on multiple factors such as stem cell source and dose (26), graft manipulation procedure (6–9), donor age (13), conditioning regimen, and post-transplant immunosuppression used. We (7, 8) and others (6, 24, 26) previously reported that immune reconstitution in children receiving TCD haploidentical transplant was characterized by early and fast NK-cell recovery constituting major lymphocyte subpopulation in the early phase following transplantation. NK-cells in the graft and fast NK-cell recovery have been considered of importance in allogeneic transplant especially on haploidentical transplant. After conditioning, blood high level of interleukin-15 provides a favorable environment not only for mature donor NK-cell infused but also for NK-cells maturing from engrafted HSCs (27). NK-cell reconstitution and its function following haploidentical transplant is clearly influenced by GvHD prophylaxis, especially by post-cyclophosphamide that eliminate most mature donor NK cells including alloreactive NK cells as it has been recently published (27).

We previously observed that the higher NK-cells numbers early post-transplant the lower incidence of relapse (8) resulted in better outcome using CD3^+^/CD19^+^ depleted grafts. We now analyzed the potential impact of CD56^dim^ and CD56^bright^ NK cells subpopulation on relapse incidence and we observed that the higher CD56^dim/^CD56^bright^ NK cell ratio, the lower relapse incidence, resulting in better DFS.

A relevant role of NK cells infused in the graft in TCD allogeneic transplant setting has been previously published (28). The number of NK cells in the graft was a powerful determinant of relapse risk. Analysis of NK cell subset clearly showed that this GvL effect was mediated by CD56^dim^ population and high-level expression of activatory receptor DNAM on CD56^dim^ population cells was also strongly protective (28). However, a similar analysis has not been published in the TCD haploidentical setting. A recent study in pediatric patients, receiving αβ^+^ T-cell depletion haploidentical transplant, showed an overshooting NK cell recovery on day +14 comparable to their donor level (29). Despite a rapid expansion of CD56^bright^ NK cell subset, CD56^dim^ NK cells were predominant during the study time period (29). However, they did not report any correlation between CD56^dim^ or CD56^bright^ recovery and relapse incidence. Our previous data indicate that the number of total NK cells early after transplantation was a strong determinant of GvL effect (8). More specifically, CD56^dim/^CD56^bright^ NK cell ratio was predictive of disease relapse and had a relevant impact on DFS.

Usually, CD56^dim^ NK cell constitute around 90% of peripheral blood NK cells in healthy donors. NK cells have a powerful activity against virus transformed cells and cancers cells specially AML cells (30, 31). This activity against cancer cells is mainly supported by CD56^dim^ cytotoxic activity (32–34). CD56^bright^ population has a different cytokine profile resulting in lower cytotoxicity against cancer cells. So, the higher CD56^dim/^CD56^bright^ NK cell ratio might result in more cytotoxicity exerted by CD56^dim^ subset and in lower risk of relapse. Unfortunately, we do not have data on cytotoxicity in our series to support this explanation. Alternatively, NK cells could act as a boost for T-cell activation that might increase the T-cell based GvL effect. However, our data clearly shows that T-cell recovery is delayed for a longer period of time following transplant especially αβ^+^ T-cells.

IL-15 is a cytokine necessary not only for NK-cell homeostasis and development but also for γδ^+^ T-cells. As during early phase following transplantation there is high levels of IL-15 in recipient, this provides a favorable environment for NK cell and γδ T-cell proliferation, maturation and function. Early reconstitution of NK and γδ^+^ T-cells comparable to their healthy donors, has been observed after allogeneic match sibling and match unrelated donors (35). In fact, we observed an association between early immune reconstitution and risk of severe infection. However, and despite rapid innate immune reconstitution there was a relevant incidence of viral reactivation as previously reported (8, 10, 11, 24). After the early post-transplant phase, CD8^+^ T-cells leaded response to infections.

T-cell recovery was delayed compared to that of NK cells, especially that of αβ^+^ T-cells. However, we observed even early after transplantation that αβ^+^ T-cells were the predominant T-cell population despite low numbers of such T-cells infused. The slow T-cell reconstitution was clearly related to the risk of opportunistic and largely viral infections. Furthermore, infections were the leading cause of NRM and the second leading cause of death after relapsing disease (Table 4).

Third, we consider our results in DFS for the whole group as acceptable taking into account the stages of disease of the study population. As expected, it was particularly good for the patients transplant in first CR. A more important result is that a relevant proportion of patients who underwent transplantation in more advanced phase of disease or even with active disease were rescued by haploidentical transplantation and still continue in CR.

The incidence of cGvHD was lower than observed using CD3^+^/CD19^+^ depletion (8). Although most patients developed mild or moderate cGvHD, near 10% of them required protracted treatment. However, cGvHD was associated with better DFS, primarily due to better control of disease relapse. Although the median follow-up is not long enough to consider the results as mature, we believe they are encouraging and support the idea of using haploidentical donors as early as possible whenever a patient needs an HSCT.

Several conclusions can be drawn. First, haploidentical transplantation using αβ^+^ TCR/CD19^+^ depletion yields encouraging results, especially in patients undergoing transplantation in early phase of disease and in complete remission. Second, the conditioning regimen used is well-tolerated. Third, ethnicity may predispose to development of severe liver toxicities what implies increased risk of NRM.

Fourth, viral infection/disease still continues being an unresolved problem clearly associated to delayed T-cell recovery that impacts on cause of death after transplant. A need for rebuilding T-cell reconstitution exits. Last but not least, rapid and sustained “mature” NK cell reconstitution results in better disease control and better transplant outcome that would support the use of fresh or IL-15 stimulated NK cell infusion early after transplant to enhance GvL with no risk of GvHD.

## Data Availability Statement

The datasets generated for this study are available on request to the corresponding author.

## Ethics Statement

Ethical review and approval was not required for the study on human participants in accordance with the local legislation and institutional requirements. Written informed consent to participate in this study was provided by the participants' legal guardian/next of kin.

## Author Contributions

MD, JZ, BM, and MG-V designed the study, analyzed the data, and wrote the manuscript. JR, JV, JS, MR, LA, AC, EG, and ES designed the study, provide and revised data, and data analysis. All authors approved final manuscript version.

### Conflict of Interest

The authors declare that the research was conducted in the absence of any commercial or financial relationships that could be construed as a potential conflict of interest.
