# Supplementary material for: Haploidentical Stem Cell Transplantation in Children With Hematological Malignancies Using αβ+ T-Cell Receptor and CD19+ Cell Depleted Grafts: High CD56dim/CD56bright NK Cell Ratio Early Following Transplantation Is Associated With Lower Relapse Incidence and Better Outcome
Source: Front Immunol. 2019 Oct 30;10:2504. doi: 10.3389/fimmu.2019.02504 (PMC6831520; doi:10.3389/fimmu.2019.02504)
Supplement: Supplementary file 1 [file Table_1.DOCX]

**Supplemental Table S1**. **Depletion procedure characteristics**

|  | CliniMacs Plus | CliniMacs Prodigy | P value |
| --- | --- | --- | --- |
| Nº procedures | 48 (72.72%) | 18 (27.27%) |  |
| T cells depletion logarithm | 4.76 (1.20-5.18) | 4.69 (4.16-9.24) | NS |
| αβ+ T cells x 10^3^ /kg | 0.00 (0.00-78.88) | 0.00 (0.00-26.32) | NS |
| CD34+ cells recovery (%9 | 83.76% (41.08-161.90) | 74.28% (37.37-112.20) | NS |
| CD34+ cells x 10^6^ /kg | 7.07 (1.02-46.62) | 9.99 (1.29-23.22) | NS |
| CD3+TCRγδ+ x 10^6^ /kg | 11.24 (0.25-83.33) | 4.67 (0.69-41.62) | 0.015 |
| B cells post (%) | 0.00 (0.00-0.04) | 0.00 (0.00-0.01) | NS |
| Granulocytes post (%) | 18.15 (0.70-64.29) | 15.60 (5.20-49.40) | NS |
| Monocytes post (%) | 60.00 (3.49-82.60) | 68.90 (42.80-88.00) | NS |
| Final viability (%) | 94.55 (65.56-99.15) | 62.14 (39.14-88.29) | 0.001 |
| CD34+ cells final viability (%) | 96.38 (89.09-99.41) | 85.00 (64.86-90.77) | 0.001 |
| Duration (minutes) | 290.00 (180-1440.0) | 1440.00 (1260-1510) | 0.001 |
|  |  |  |  |

**Supplemental Table S2. Details on lymphocytes subpopulations, median (range)**

|  | **1 month** | **3 months** | **6 months** | **9 months** | **12 months** |
| --- | --- | --- | --- | --- | --- |
| CD3+ cells/μL | 155 (11-1694) | 293 (5-6250) | 490 (18-3250) | 667 (39-4320) | 909 (14-4352) |
| CD4+ cells/μL | 25 (0-429) | 83 (0-1204) | 178 (4-895) | 275 (2-2400) | 437 (55-2598) |
| CD8+ cells/μL | 26 (1-1181) | 82 (0-4927) | 165 (1-2690) | 247 (5-1478) | 309 (31-1478) |
| αβ+ T cells/μL | 40 (1-1360) | 156 (0-5470) | 398 (10-3035) | 571 (5-4056) | 854 (75-4056) |
| γδ+ T cells/μL | 32 (0-573) | 50 (0-1883) | 86 (2-470) | 74 (1-663) | 95 (19-340) |
| CD19+ cells/μL | 2 (0-414) | 90 (1-4927) | 240 (1-999) | 357 (2-1373) | 493 (4-1414) |
| CD3-CD56+ cells/μL | 250 (1-2046) | 235 (20-1380) | 185 (8-1381) | 198 (39-658) | 166 (33-756) |
