# Supplementary material for: Haploidentical Stem Cell Transplantation in Children With Hematological Malignancies Using αβ+ T-Cell Receptor and CD19+ Cell Depleted Grafts: High CD56dim/CD56bright NK Cell Ratio Early Following Transplantation Is Associated With Lower Relapse Incidence and Better Outcome
Source: Front Immunol. 2019 Oct 30;10:2504. doi: 10.3389/fimmu.2019.02504 (PMC6831520; doi:10.3389/fimmu.2019.02504)
Supplement: Supplementary file 2 [file Image_1.pdf]

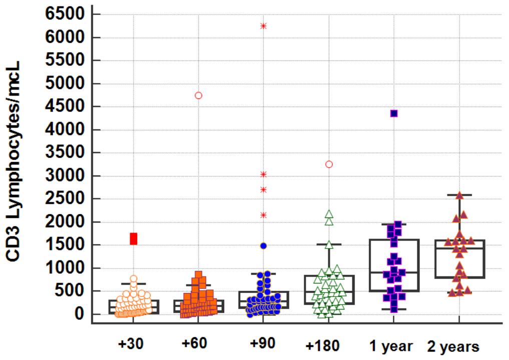

A.

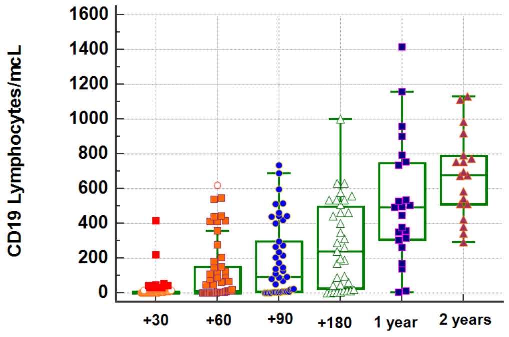

B.

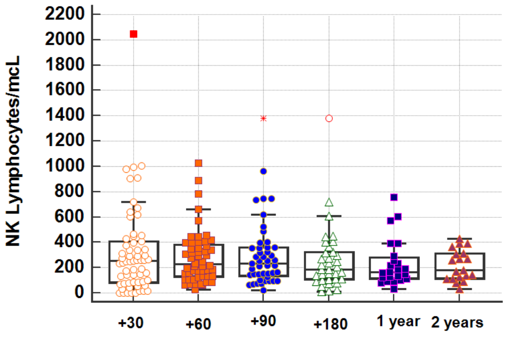

C.

Supplemental Figure S1 legend. Immune Reconstitution Kinetics at day +30, +60, +90, +180, +1 year and +2 years post-transplantation. A, CD3 lymphocytes; B, CD19 lymphocytes; C, NK cells.
